# The effect of daytime napping on the athletic performance of team ball sports athletes: systematic review and meta-analysis

**DOI:** 10.3389/fphys.2026.1878224

**Published:** 2026-07-22

**Authors:** JiaNing Zheng, YiFan Ye, Xiaotian Li, Jiaxin Peng

**Affiliations:** 1 Department of Physical Education, Central South University, Changsha, China; 2 Wuhan Sports University, Wuhan, China

**Keywords:** agility, athletic recovery, daytime napping, endurance, meta-analysis, sleep, sports performance, team sports

## Abstract

This systematic review and meta-analysis aimed to estimate the effects of daytime napping on athletic performance across multiple domains in team ball sports athletes. Five electronic databases (PubMed, Scopus, Web of Science, Cochrane Library, and SPORTDiscus) were systematically searched from their inception to December 18, 2025, to identify randomized controlled trials (RCTs) investigating the association between daytime napping and objective performance measures compared with a no-nap control condition. Twelve trials encompassing 179 team ball sports athletes met the inclusion criteria. Nap interventions were consistently scheduled in the early afternoon (13:00–15:30), most frequently commencing at 14:00. Following presumed normal nocturnal sleep, napping demonstrated a large positive effect on endurance performance (SMD = 0.96, 95% CI 0.62 to 1.29, p < 0.001) and a moderate improvement in agility (SMD = 0.55, 95% CI 0.01 to 1.09, p = 0.048). A moderate positive trend was also observed for power and strength (SMD = 0.50, 95% CI -0.09 to 1.09), although the 95% confidence intervals crossed zero. Subgroup analyses revealed that the magnitude of these effects was modulated by nap duration, with longer naps (>60 min) yielding the largest improvements in endurance (SMD = 1.38) and power (SMD = 0.84), while moderate naps (31–60 min) similarly conferred substantial benefits. These findings suggest that strategic napping, particularly moderate and long durations, should be considered for integration into training and competition routines for athletes achieving presumed normal nocturnal sleep. However, effects on cognitive and technical performance remain uncertain due to evidence imprecision. Strategic napping protocols should be tailored to specific sport performance demands to optimize physiological recovery and readiness.

**Systematic Review Registration:**

https://www.crd.york.ac.uk/prospero/display_record.php?ID=CRD420251268837, identifier CRD420251268837.

## 1 Introduction

It is well established that nocturnal sleep of sufficient duration and quality is essential for optimal athletic performance (Walsh et al., 2021). For team ball sports athletes (e.g., those in soccer, basketball, volleyball, handball, and rugby), the physiological and cognitive demands of training and competition are exceptionally high (Clemente et al., 2025). These athletes must repeatedly execute complex motor skills, make rapid decisions under pressure, sustain high-intensity intermittent effort, and remain alert throughout matches that last 60 to 90 minutes or longer (Cortez et al., 2026). Crucially, each of these performance capabilities is highly sensitive to sleep loss. Even modest reductions in sleep duration may impair reaction time, decision-making accuracy, sprint performance, and technical skill execution (Wannell et al., 2025; Lopes et al., 2022; Craven et al., 2022).

Despite the well-documented importance of sleep, team ball sports athletes frequently encounter environmental and scheduling factors that compromise their nocturnal sleep. These include pre-competition anxiety and arousal, travel across time zones causing jet lag, late evening training or matches, and early morning travel for away fixtures (Reilly, 2009; Fullagar et al., 2015; O’Donnell et al., 2018; van Rensburg et al., 2021). In such contexts, daytime napping emerges as a practical, non-pharmacological strategy to mitigate sleep debt and enhance overall recovery (Mesas et al., 2023; Boukhris et al., 2023a).

Daytime napping serves two complementary purposes. First, it can compensate for partial sleep deprivation incurred during the previous night, restoring alertness and attenuating performance decrements (Mesas et al., 2023). Second, recent evidence suggests that daytime napping may confer additional ergogenic benefits even following a presumed normal night’s sleep. Physiologically, this may be achieved by reducing accumulated homeostatic sleep pressure, promoting parasympathetic autonomic dominance, and facilitating the clearance of metabolic byproducts within the central nervous system (Xie et al., 2013; Charest and Grandner, 2020; Botonis et al., 2021; Xu et al., 2025).

The ergogenic effects of napping are likely modulated by several interacting factors. These variables include nap duration and timing, the interval between nap awakening and subsequent performance (i.e., to account for sleep inertia), an individual’s habitual napping pattern, and the specific physiological demands of the sport (Mesas et al., 2023; Bensalem et al., 2025; Öncü et al., 2026). Among these factors, nap duration is arguably the most critical and practically modifiable, with potential differences between short (≤30 min), moderate (31–60 mi*n*), and long (>60 min) naps in terms of sleep architecture, recovery of different physiological systems, and susceptibility to sleep inertia upon awakening (Mesas et al., 2023; Patterson et al., 2021). Crucially, while daytime napping is widely utilized for its restorative properties, it must be acknowledged that napping may have both beneficial and potentially adverse effects depending on its duration and timing. While short to moderate naps are generally considered highly restorative, excessively long daytime naps may increase intradaily variability and contribute to a greater fragmentation of the circadian rest-activity rhythm. In this regard, current recommendations from sleep authorities, such as the National Sleep Foundation, generally discourage prolonged daytime napping to maintain robust circadian alignment. Therefore, the ergogenic effects of napping must be carefully interpreted within the broader context of circadian rhythm regulation.

Athletic performance in team ball sports is inherently multidimensional (Sarmento et al., 2022). A comprehensive evaluation of the existing literature reveals that investigators assess athletic performance employing a highly diverse array of outcome measures. Based on a systematic synthesis of these measures, we identified five distinct domains that collectively capture the essential performance requirements of team ball sports athletes: (1) Cognitive and Attention Metrics (e.g., reaction time, vigilance, D2 attention test performance) (Boukhris et al., 2020; Teece et al., 2023; Eken et al., 2024), (2) Agility Metrics (e.g., T-test time, shuttle run distance) (Eken et al., 2024; Nishida et al., 2021; Eken et al., 2025; Souabni et al., 2023a), (3) Power and Strength Metrics (e.g., countermovement jump height, peak power output) (Xu et al., 2025; Boukhris et al., 2020; Teece et al., 2023; Eken et al., 2024; Souabni et al., 2023a), (4) Endurance and Distance Metrics (e.g., Yo-Yo intermittent recovery test performance, total distance covered) (Xu et al., 2025; Boukhris et al., 2020; Hsouna et al., 2022; Boukhris et al., 2023b, Boukhris et al., 2023c, Boukhris et al., 2024), and (5) Technical Skill Metrics (e.g., shooting or passing accuracy) (Nishida et al., 2021; Souabni et al., 2023b). The relative contribution of each domain inherently varies across sporting disciplines; for instance, endurance capacity is paramount in soccer, whereas power and agility are arguably more decisive in basketball and volleyball (Nishida et al., 2021; Souabni et al., 2023b; Morrison et al., 2022). Consequently, estimating the effects of napping on each domain separately is essential for developing sport-specific recommendations.

Several systematic reviews have synthesized the qualitative evidence on napping and athletic performance. Recent qualitative reviews have reported that napping appears to improve physical performance outcomes (e.g., jump height, sprint speed) and cognitive function (e.g., attention, reaction time) while simultaneously reducing perceptions of fatigue (Mesas et al., 2023; Dolezal et al., 2017; Souabni et al., 2021). Narrative reviews have similarly highlighted the potential of napping as a recovery strategy in sport settings (Dolezal et al., 2017; Schmidt et al., 2025).

However, to the best of our knowledge, no meta-analysis has yet quantified the magnitude of these effects specifically in team ball sports athletes. Crucially, this review addresses a major conceptual gap in the existing literature. Previous systematic reviews have predominantly aggregated divergent athletic tasks into a generic “physical performance” category, which masks domain-specific ergogenic effects. In contrast, the structural and conceptual novelty of our work lies in systematically deconstructing performance into five distinct domains—cognitive, agility, power, endurance, and technical skill—tailored specifically to the unique bioenergetic and neuromuscular demands of team sports.

Therefore, the objective of this systematic review and meta-analysis was to synthesize evidence from randomized controlled trials and estimate the standardized mean difference (SMD) of daytime napping across these five conceptually defined domains. Furthermore, this study fills a critical gap by quantitatively comparing the dose-response effects of different nap durations (short: ≤30 minutes; moderate: 31–60 minutes; long: >60 minutes) to provide actionable, sport-specific recommendations.

## 2 Materials and methods

### 2.1 Protocol registration

This systematic review followed the Preferred Reporting Items for Systematic Reviews and Meta-Analyses (PRISMA) guidelines (Page et al., 2021) and the PERSiST guidance (Ardern et al., 2022). The PRISMA checklist is available in the **Supplementary Material 2**. The protocol for this review was registered in the PROSPERO database for systematic reviews (CRD420251268837).

### 2.2 Information sources and search strategy

A systematic literature search was conducted across five electronic databases: PubMed, Scopus, Web of Science, Cochrane Library, and SPORTDiscus (via EBSCOhost). The objective was to identify randomized controlled trials (RCTs) investigating the association between daytime napping and athletic performance or fatigue in sports contexts. The search encompassed all records from database inception until 18 December 2025, without any language or publication date restrictions at the initial screening stage.

### 2.3 Eligibility criteria

The eligibility criteria were defined according to the PICO(S) framework as follows:

1. Participants: Healthy human athletes participating in team ball sports (e.g., soccer, basketball, volleyball, handball, hockey, rugby, water polo, ice hockey). There were no restrictions on age, sex, or competitive level (elite, amateur, and student athletes were eligible). (2) Intervention: Any form of planned, intentional nap undertaken during the daytime (typically between noon and evening) and intended to be restorative. No restrictions were placed on nap duration, timing (e.g., post-lunch, pre-training), or environmental conditions. (3) Comparison: A no-nap control condition, including either maintenance of wakefulness during usual quiet activities (e.g., reading) or supine rest while remaining awake (i.e., quiet rest). (4) Outcomes: At least one objective, quantitative measure of athletic performance or cognitive function was required. Considering that sleep loss exerts heterogeneous physiological impacts across various bioenergetic and neuromuscular systems (Walsh et al., 2021), we prospectively deconstructed athletic performance into five distinct domains to ensure higher intra-domain homogeneity: (1) Cognitive and Attention Metrics (e.g., reaction time, attention tests such as the D2),
2. Agility Metrics (e.g., agility T-test, shuttle run distance), (3) Power and Strength Metrics (e.g., jump height, peak power output), (4) Endurance Metrics (e.g., Yo-Yo test performance, total distance), and (5) Technical Skill Metrics (e.g., shooting/passing accuracy). This taxonomy acknowledges the system-specific vulnerability to sleep restriction and aims to mitigate the methodological risk of aggregating functionally divergent outcomes (Walsh et al., 2021; Higgins et al., 2022). (5) Study design: Randomized controlled trials (RCTs), including both parallel-group and crossover designs with adequate washout periods.

The exclusion criteria were as follows: (1) studies involving individual sport athletes, non-athletic populations, or animal subjects; (2) evaluations of interventions exclusively targeting nocturnal sleep (e.g., sleep extension), or used pharmacological/device-assisted sleep induction; (3) lack of a no-nap control group or used an alternative nap intervention as the sole comparator; (4) reporting of only subjective scales (e.g., fatigue ratings) or physiological biomarkers without a linked objective performance outcome; (5) non-RCTs (e.g., observational studies, case reports), reviews, meta-analyses, conference abstracts, or editorials.

### 2.4 Selection process and data collection

The search strategy integrated terms related to daytime napping (e.g., “nap”, “daytime nap”, “afternoon nap”, “power nap”, “siesta”) with terms pertaining to sports disciplines, athletes, and key performance outcomes (e.g., “soccer”, “football”, “basketball”, “athlete”, “player”, “performance”, “endurance”, “sprint*”, “fatigue”, “cognitive performance”). The complete search syntax for all databases is detailed in the **Supplementary Table 1**. To ensure comprehensive coverage, the reference lists of all included studies and relevant review articles, along with their citing publications, were manually screened for additional eligible records. All records identified from the database searches were imported into EndNote (version X9, Clarivate Analytics, Pennsylvania, USA) for deduplication.

Study selection was conducted independently by two reviewers (JiaNing Zheng [JNZ] and Yifan Ye [YFY]), with disagreements resolved through discussion to reach a consensus. The selection process involved two stages: first, screening of titles and abstracts against the eligibility criteria, and second, a detailed full-text assessment of potentially eligible studies for final inclusion.

For each included study, data were systematically extracted by one reviewer (JNZ) and independently verified by a second reviewer (YFY). The extracted information included: (1) study characteristics; (2) participant details; (3) intervention protocol (e.g., nap duration, timing); (4) control condition; (5) outcome measures; and (6) key results. In instances where numerical data were presented exclusively within figures, relevant values were extracted utilizing the PlotDigitizer online software (https://plotdigitizer.com) (Eken et al., 2025; Nishida et al., 2021; Eken et al., 2024; Hsouna et al., 2022). Contacting original authors for additional data was not required.

### 2.5 Risk of bias assessment

The risk of bias for each included randomized controlled trial was independently assessed by two reviewers (JNZ and YFY) using the Cochrane Collaboration’s revised tool for assessing risk of bias in randomized trials (RoB 2.0) (Sterne et al., 2019). Any disagreement between reviewers was resolved through discussion. The RoB 2.0 tool evaluates bias across five specific domains: (1) bias arising from the randomization process, (2) bias due to deviations from intended interventions, (3) bias due to missing outcome data, (4) bias in the measurement of the outcome, and (5) bias in the selection of the reported result. Each domain was judged as “low risk,” “some concerns,” or “high risk” based on the tool’s signaling questions and criteria. An overall risk-of-bias judgment for each study was derived according to the RoB 2.0 algorithm: a study was classified as “low risk” if all domains were judged as low risk; as having “some concerns” if at least one domain raised some concerns (but none were high risk); and as “high risk” if at least one domain was judged as high risk or if multiple domains raised concerns that substantially compromised the result’s credibility. The results of this assessment are presented in a risk-of-bias summary figure.

### 2.6 Synthesis methods

Given the primary aim of assessing the effect of daytime napping on athletic performance in team ball sports, we employed a two-stage synthesis approach. First, to address the diversity of specific performance metrics, all outcomes were synthesized within five pre-defined performance domains: (1) Cognitive and Attention Metrics (e.g., reaction time, attention tests such as the D2), (2) Agility Metrics (e.g., agility T-test, shuttle run distance), (3) Power and Strength Metrics (e.g., jump height, peak power output), (4) Endurance Metrics (e.g., Yo-Yo test performance, total distance), and (5) Technical Skill Metrics (e.g., shooting/passing accuracy). Separate meta-analyses were conducted for each domain.

Second, to investigate the influence of nap duration, a secondary analysis was performed on the pooled effect across all performance domains. This overall estimate was then stratified into three nap duration subgroups: short naps (≤30 minutes), moderate naps (31–60 minutes), and long naps (>60 minutes). In studies that investigated multiple nap durations, each duration was analyzed as an independent comparison against the common no-nap control group. For studies reporting separate data for subgroups under identical experimental conditions, these data were combined to create a single study-level estimate prior to inclusion in the meta-analysis. For instance, in a study involving elite volleyball players, countermovement jump height, volleyball agility test performance, and D2 attention test results were reported separately for male and female participants; therefore, these data were pooled to derive a combined estimate for each outcome (Eken et al., 2024).

For studies reporting data at multiple time points, post-intervention measurements taken closest to the nap period were selected for the primary analysis to best capture the immediate effect of the nap. As an example, one study compared peak power (6-second cycle test), reaction time, fatigue, muscle soreness, and alertness between nap and control groups at both morning (pre-nap) and afternoon (post-nap) time points; only the afternoon (post-nap) data were included in our main synthesis (Teece et al., 2023). This decision aligns with the intervention’s acute purpose and ensures consistency with other included studies that primarily assessed outcomes following the nap opportunity.

However, it should be noted that one study differed from the others in a more fundamental aspect: participants underwent a simulated late-evening soccer match (21:00) involving the Loughborough Intermittent Shuttle Test on the night prior to the nap intervention, whereas the remaining studies did not report any evening exercise protocol (Hsouna et al., 2022). Given that residual fatigue from prior evening exercise may attenuate the subsequent ergogenic effect of napping, we pre-specified a sensitivity analysis excluding this study to assess its influence on the pooled estimates.

The standardized mean difference with 95% confidence intervals (CIs) was selected as the summary effect measure due to the variety of assessment scales used across studies. For continuous outcomes, initial SMDs were calculated prior to RVE implementation using the standard inverse variance method (random-effects model) via RevMan 5.3 software. These initial unadjusted calculations utilized the post-intervention means, standard deviations, and sample sizes from the nap and control groups, as exact within-participant correlations or change-scores were rarely reported in the primary crossover trials. When studies reported outcomes as mean ± standard error (SE) or median (range), the mean and standard deviation (SD) were estimated using established methods. Prior to meta-analysis, we assessed the tenability of the normality assumption for continuous outcome data. We examined whether primary studies reported formal tests of normality (e.g., Shapiro-Wilk test). Studies flagged for skewness were retained in the primary analysis, but their influence on the pooled estimates was examined in leave-one-out sensitivity analyses.

To facilitate interpretability and enable meaningful comparisons across performance domains, all effect sizes were standardized such that a positive SMD consistently indicates improved athletic performance. For outcomes where lower values represent better performance (e.g., time-based agility tests such as the T-test or shuttle run time), the individual effect sizes were multiplied by -1 prior to meta-analytic pooling. This transformation ensures that across all five domains, a positive SMD uniformly denotes performance enhancement. The direction of transformation for each outcome was explicitly recorded and verified independently by both authors (JNZ and YFY).

Because several studies contributed more than one effect size to the same performance domain (e.g., peak power and average power in the Power domain; higher distance and total distance in the Endurance domain), these outcomes are statistically dependent as they derive from the same participants. To avoid arbitrary selection of a single outcome or loss of information through naive aggregation, we employed robust variance estimation (RVE) (Hedges et al., 2010). RVE adjusts the standard errors for clustering of effect sizes within studies and does not require knowledge of the exact within-study correlations, which are rarely reported. All analyses were conducted in R (version 4.5.2; R Core Team, 2025) using the robumeta (Fisher et al., 2017) and clubSandwich (Pustejovsky, 2024) packages. For each performance domain, we fitted an intercept-only model with study ID as the clustering variable. A correlated effects weighting scheme was used with a within-study correlation assumption of ρ = 0.7, representing a moderate-to-strong correlation between related outcomes. Small-sample corrections were applied using the clubSandwich method to provide robust CIs with improved coverage (Pustejovsky and Tipton, 2018). Sensitivity analyses were performed by varying the assumed within-study correlation (ρ = 0.2, 0.5, 0.8). Subgroup analyses based on nap duration (short: ≤30 min; moderate: 31–60 min; long: >60 min) were conducted by restricting the dataset to each subgroup and re-applying RVE.

Between-study heterogeneity was quantified using the τ² statistic (Higgins et al., 2003). To further explore potential sources of heterogeneity, meta-regression was considered. However, because the number of studies contributing to each performance domain was less than 10, meta-regression was not performed, as this method is not recommended when fewer than 10 studies are available (Deeks et al., 2022). This decision aligns with methodological guidelines.

In accordance with recent recommendations emphasizing estimation over dichotomous testing, we report effect sizes with 95% confidence intervals as the primary basis for inference. We refrain from using the term ‘statistically significant’ and instead interpret findings based on the magnitude, direction, and precision of the effect estimates. A two-sided p-value <0.05 was considered as **Supplementary Information**. For each pooled estimate, we report the Satterthwaite degrees of freedom (df) obtained from the clubSandwich small−sample correction, as this df directly reflects the effective information available for inference.

Sensitivity analyses were performed to assess the robustness of the pooled estimates. These included: (i) the leave-one-out analysis; (ii) the pre-specified subgroup analyses by nap duration; and (iii) a pre-planned sensitivity analysis excluding one study that involved a unique evening exercise protocol (Hsouna et al., 2022), as described above. A sensitivity analysis stratifying studies by risk of bias (RoB) category was not conducted for the following methodological reasons: (1) the methodological quality across studies was relatively homogeneous, with the majority exhibiting similar RoB profiles; (2) the number of studies judged to be at ‘high risk’ of bias was very small (n<3), meaning that a stratified analysis would be underpowered and could yield unstable or misleading estimates; and (3) the consistency of results across the leave-one-out and subgroup analyses suggested that the overall findings were not unduly influenced by any single study or particular study characteristic.

## 3 Results

### 3.1 Study selection

**Figure 1:**
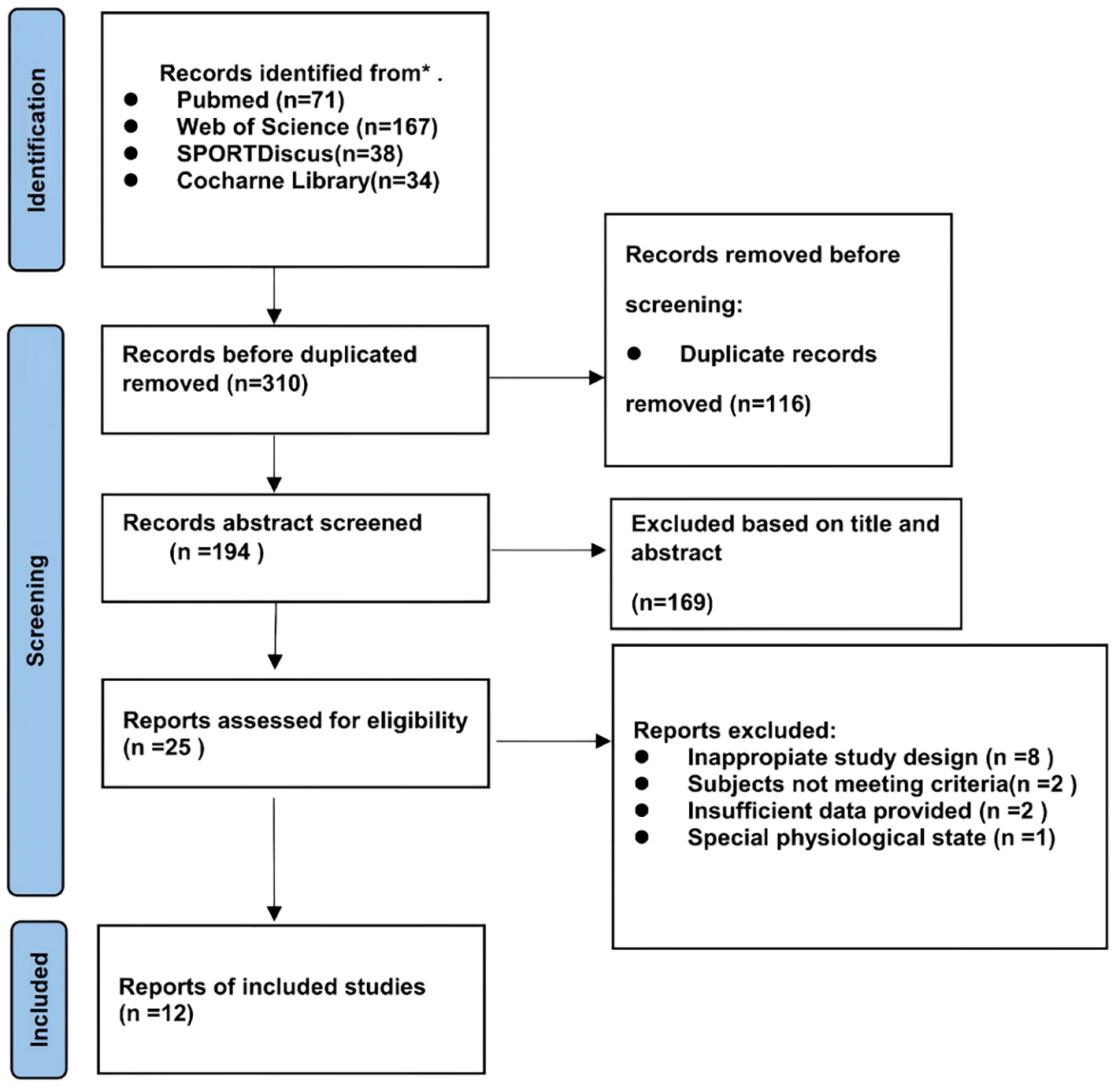
Flow diagram of the study selection.

As illustrated in **Figure 1**, of the 310 initially identified studies, 25 were selected for full-text assessment, of which 13 were excluded for not meeting the inclusion criteria. A complete list of excluded studies with specific reasons is provided in the **Supplementary Table 2**. Thus, 12 studies were finally included (Xu et al., 2025; Boukhris et al., 2020; Teece et al., 2023; Eken et al., 2024; Nishida et al., 2021; Eken et al., 2025; Souabni et al., 2023a; Hsouna et al., 2022; Boukhris et al., 2023a, Boukhris et al., 2023b, Boukhris et al., 2024; Souabni et al., 2023b).

### 3.2 Characteristics of the included studies

The characteristics and main findings of the included studies are summarized in **Table 1**. Nearly half of the studies (n = 5) were conducted in Tunisia (Boukhris et al., 2020; Hsouna et al., 2022; Boukhris et al., 2023a, Boukhris et al., 2023b, Boukhris et al., 2024), two in France (Souabni et al., 2023a, Souabni et al., 2023b), one in New Zealand (Teece et al., 2023), one in Japan (Nishida et al., 2021), two in Turkey (Eken et al., 2024, Eken et al., 2025), and one in China (Xu et al., 2025). Sample sizes ranged from 10 to 25 participants per trial, yielding a total of 179 healthy team ball sports athletes (including 84 soccer players, 29 rugby players, 28 handball players, 22 basketball players, and 16 volleyball players). Sleep parameters were assessed using actigraphy or accelerometry in some studies (Teece et al., 2023; Hsouna et al., 2022; Boukhris et al., 2023a, Boukhris et al., 2023b, Boukhris et al., 2024; Souabni et al., 2023b), while others reported that habitual nocturnal sleep typically lasted 7−9 h (Boukhris et al., 2020; Souabni et al., 2023a). Several studies utilized self-reported measures to record nap duration (Eken et al., 2024, Eken et al., 2025), while one employed the Pittsburgh Sleep Quality Index to verify generally good sleep quality among participants (Xu et al., 2025).

**Table 1:** Characteristics of the randomized controlled trials.

Authors, year	Journal title	Country	Study population*	Sample size	Mean age (year) ± SD	Napping start time^‡^	Nap duration groups (min)	Wash-out	Sleep assessment	Outcome analyzed and main results^§^				
										Cognitive & attention	Agility	Power & strength	Endurance & distance	Technical skill
Souabni et al., 2023a	Research in Sports Medicine	France	Male, Basketball	12	26.33 ± 5.2	13:00	0, 40	3 days	SR	(-)	↑*SMD*: 0.42-0.60 (40 min)	↔*SMD*: 0.85 [-0.36, 2.06] (40 min)	(-)	(-)
Boukhris et al., 2020	International Journal of Environmental Research and Public Health	Tunisia	Male, Football, rugby, handball	14	20.3 ± 3.0	14:00	0, 40, 90	3 days	SR	↔*SMD*: 0.51-0.65 (40/90 min)	(-)	↔*SMD*: 0.41-0.71 (40/90 min)	↔*SMD*: 0.91-1.59 (40/90min)	(-)
Hsouna et al., 2022	Research in Sports Medicine	Tunisia	Male, Football, rugby, handball	12	23 ± 3	14:00	0, 40	3 days	ACT	(-)	(-)	(-)	↑ *SMD*: 1.23-1.24 (40 min)	(-)
Teece et al., 2023	Sleep	New Zealand	Male, Rugby	15	21 ± 2	14:00	0, 60	7 days	ACT	↔*SMD*: -0.12 [-0.84, 0.60] (60 min)	(-)	↔*SMD*: 0.09-0.18 (60 min)	(-)	(-)
Nishida et al., 2021	Sports Medicine International Open	Japan	Male, Handball	11	20.7 ± 1.2	13:00	0, 20, 60	7 days	ACT	(-)	↑*SMD*: 0.08-0.09 (20/60min)	(-)	(-)	↔*SMD*: 0.09 [-1.10,1.28] (20/60 min)
Xu et al., 2025	Nature and Science of Sleep	China	Male, Football	25	20 ± 1	13:00 (30), 14:00 (90)	0, 30, 90	3 days	PSQI	(-)	(-)	↑*SMD*: 0.72-1.12 (30/90min)	↑*SMD*: 1.04-1.32 (30/90 min)	(-)
Souabni et al., 2023b	Biology of Sport	France	Male, Basketball	10	27.6 ± 4.7	13:00	0, 40	3 days	ACT	(-)	(-)	(-)	(-)	↑*SMD*: 1.47-1.95 (40 min)
Boukhris et al., 2023a	Research in Sports Medicine	Tunisia	Male, Football, rugby, handball	15	20 ± 3	14:00	0, 40	3 days	ACT	(-)	(-)	(-)	↑*SMD*: 1.07-1.43 (40 min)	(-)
Boukhris et al., 2023b	Research in Sports Medicine	Tunisia	Male, Football, rugby, handball	16	20 ± 3	14:00	0, 40, 90	3 days	ACT	(-)	(-)	(-)	↔*SMD*: 1.00-1.65 (40/90 min)	(-)
Eken et al., 2024	Medicina	Turkey	8 Males, 8 Females, Volleyball	16	23 ± 3	13:15	0, 25, 45	7 days	SR	↑*SMD*: 3.73-5.85 (25/45 min)	↑*SMD*: 0.75-0.84 (25/45min)	↔*SMD*: -0.01-0.11 (25/45 min)	(-)	(-)
Eken et al., 2025	Life	Turkey	Male, Football, rugby, handball	16	17.18 ± 1.04	14:00	0, 25, 60	2 days	SR	(-)	↑*SMD*: 0.36-1.09 (25/60min)	(-)	(-)	(-)
Boukhris et al., 2024	Journal of Sleep Research	Tunisia	Male, Football, rugby, handball	17	20 ± 3	14:00	0, 40	3 days	ACT	(-)	(-)	(-)	↑*SMD*: 1.03-1.31 (40 min)	(-)

* All study participants were primarily male athletes, while data from female participants is relatively limited. Sport classifications reflect the specific sporting cohorts investigated in the respective trials.

‡ Data available for daytime napping taken at 13:00 to 15:30 were used in the present analyses.

§ The signals in the outcomes analyzed and main results columns indicate:

(↑) Positive effect with 95% CI entirely above zero; (↔) Positive point estimate with 95% CI crossing zero (compatible with null or small negative effects); (-) Outcome not assessed. *SMD*, Standardized Mean Difference.

ACT, accelerometer; PSQI, Pittsburgh Sleep Quality Index; SR, self-reported

All studies employed a randomized crossover design, with washout periods between nap and no-nap conditions ranging from 2 to 7 days. Napping was consistently scheduled in the early afternoon (range *13:00–15:30*), with 14:00 being the most common start time. Regarding nap duration, six studies (Xu et al., 2025; Boukhris et al., 2020; Eken et al., 2024; Nishida et al., 2021; Boukhris et al., 2023b; Eken et al., 2025) examined two or more durations spanning 20 to 90 min, while the remaining studies employed a single duration of 31−60 min. Cognitive performance was primarily assessed using digit cancellation tests or reaction time tasks. Endurance performance was most frequently evaluated using the 5 m shuttle run test.

### 3.3 Main results from the meta-analyses

A total of 49 effect sizes from 12 studies were included, covering five performance domains. Robust variance estimation (RVE; ρ = 0.7) was used to account for dependent effect sizes.

**Figure 2:**
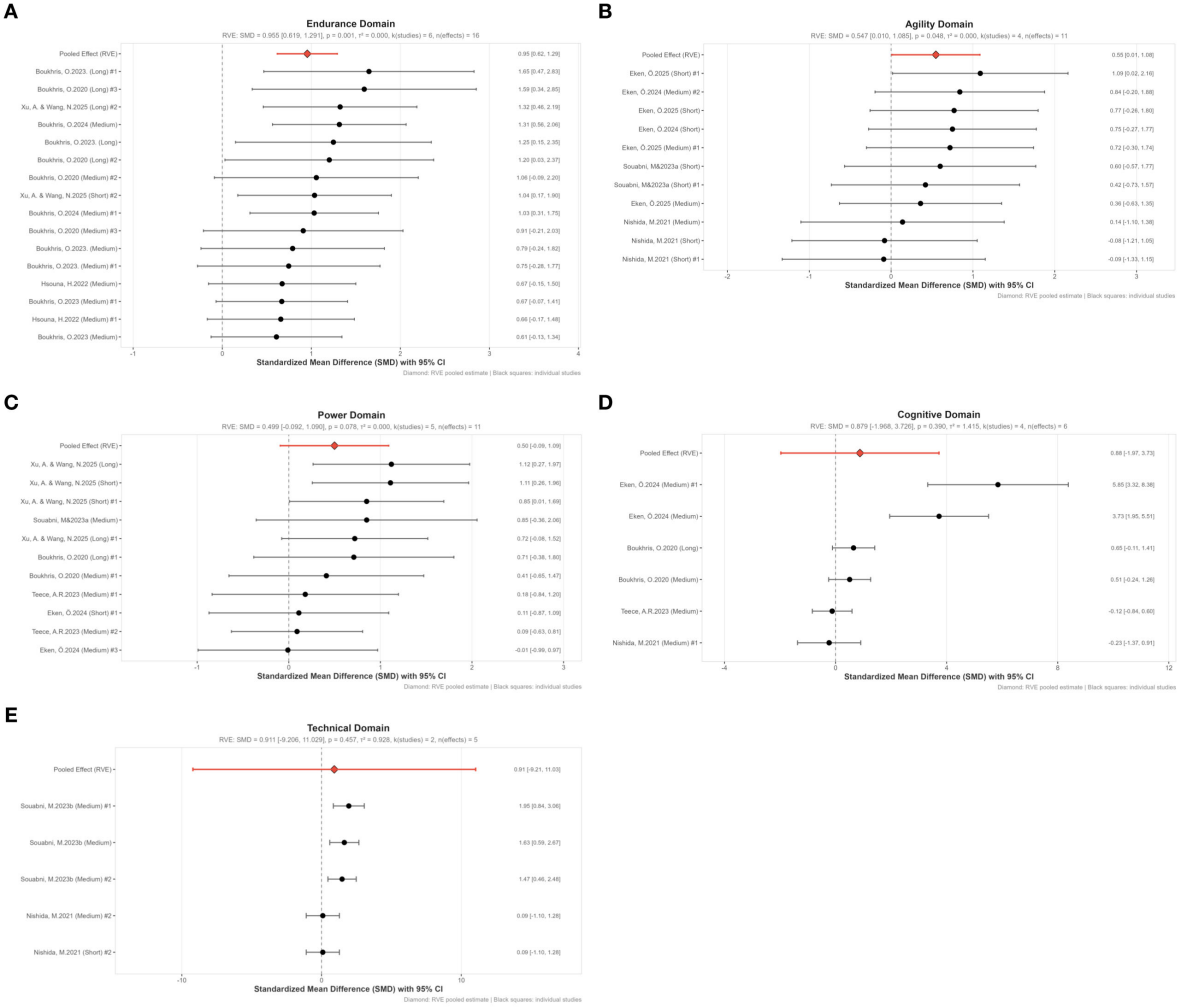
**(A)** Forest plot for endurance performance. **(B)** Forest plot for agility performance. **(C)** Forest plot for power and strength performance. **(D)** Forest plot for cognitive performance. **(E)** Forest plot for technical skill performance. The wide confidence interval reflects substantial heterogeneity arising from diverse technical skill measures (e.g., single shooting accuracy vs. composite performance indices).

Napping exerted a large beneficial effect on endurance performance, with the 95% confidence interval lying entirely above zero (*SMD =* 0.96, 95% CI 0.62 to 1.29, *p <* 0.001, *df =* 4.44) (**Figure 2A**). Regarding agility, after standardizing effect sizes such that positive values indicate improved performance, the pooled SMD was 0.55 (95% CI 0.01 to 1.09, *p =* 0.048, *df =* 2.93) (**Figure 2B**), indicating that napping enhanced agility performance. For power and strength metrics, the pooled effect size suggested a moderate positive trend (*SMD =* 0.50, 95% CI -0.09 to 1.09, *p =* 0.078, *df =* 3.71) (**Figure 2C**). However, the 95% confidence interval crossed zero, indicating that the data are compatible with a negligible negative effect, no effect, or a large positive effect. Therefore, definitive conclusions regarding the ergogenic effect of napping on power and strength performance cannot be drawn from the current evidence. In contrast, evidence regarding the cognitive (*SMD =* 0.88, 95% CI -1.97 to 3.73, *p =* 0.390, *df =* 2.86) (**Figure 2D**) and technical (*SMD =* 0.91, 95% CI -9.21 to 11.03, *p =* 0.457, *df =* 1.00) (**Figure 2E**) domains did not reliably indicate beneficial effects, as the extremely wide confidence intervals reflected substantial imprecision. Between-study heterogeneity was observed in the cognitive (τ² = 1.42) and technical (τ² = 0.93) domains, whereas heterogeneity was absent (τ² = 0) in the remaining domains.

**Figure 3:**
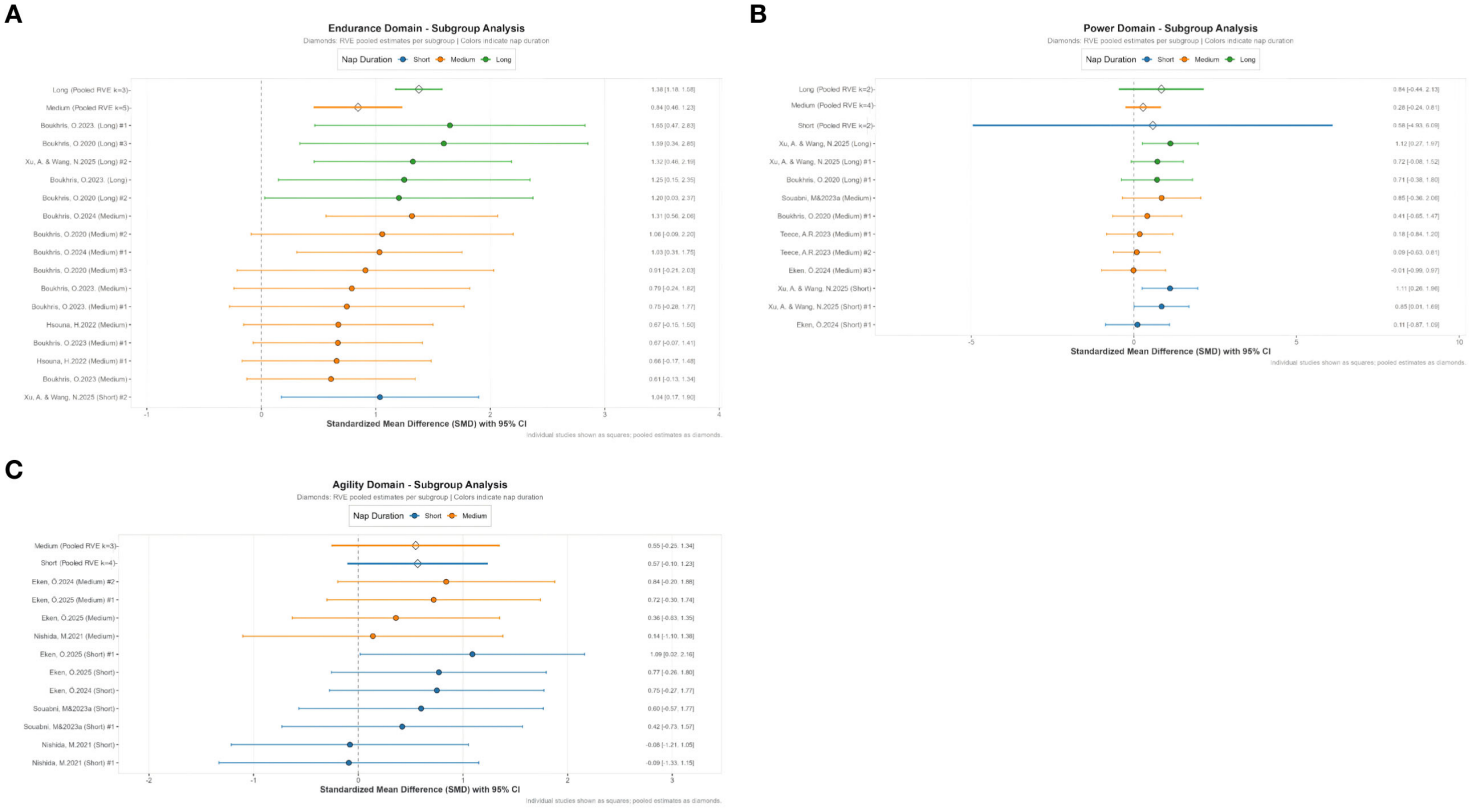
**(A)** Forest plot for subgroup analysis of endurance performance by nap duration. **(B)** Forest plot for subgroup analysis of power and strength performance by nap duration. **(C)** Forest plot for subgroup analysis of agility performance by nap duration.

Subgroup analyses stratified by nap duration were performed for each domain. Given the small number of studies in some subgroups, these estimates should be interpreted cautiously. RVE-corrected results (ρ = 0.7) by nap duration were as follows:

Within the endurance domain, both moderate (*SMD =* 0.85, 95% CI 0.46 to 1.23, *p* = 0.004, *df =* 3.55) and long (*SMD =* 1.38, 95% CI 1.18 to 1.58, *p* = 0.002, *df =* 1.83) naps yielded improvements, with confidence intervals lying entirely above zero, providing evidence of beneficial effects (**Figure 3A**). For power, while short and moderate naps yielded small to moderate point estimates (short: *SMD =* 0.58; moderate: *SMD =* 0.29), long naps produced the largest point estimate (*SMD =* 0.84, 95% CI -0.44 to 2.13, *p* = 0.076, *df =* 1.00). However, all wide confidence intervals crossed zero, reflecting the limited number of contributing studies (e.g., k = 2 for long naps); thus, while the point estimate suggested a positive trend, definitive conclusions regarding the effect of long naps on power performance cannot be drawn from the current evidence (**Figure 3B**). In the agility domain, after direction standardization, both short and moderate naps showed positive SMDs (short: *SMD =* 0.57, 95% CI -0.10 to 1.23, *p* = 0.072,d*f =* 2.94; moderate: *SMD =* 0.55, 95% CI -0.25 to 1.34, *p* = *0.096, df =* 1.93), indicating improved agility performance (**Figure 3C**). However, the confidence intervals for both estimates crossed zero, reflecting the limited number of studies in these subgroups. No clear effects were observed for cognitive or technical domains across any nap duration, partly owing to limited data.

### 3.4 Sensitivity analysis

**Figure 4:**
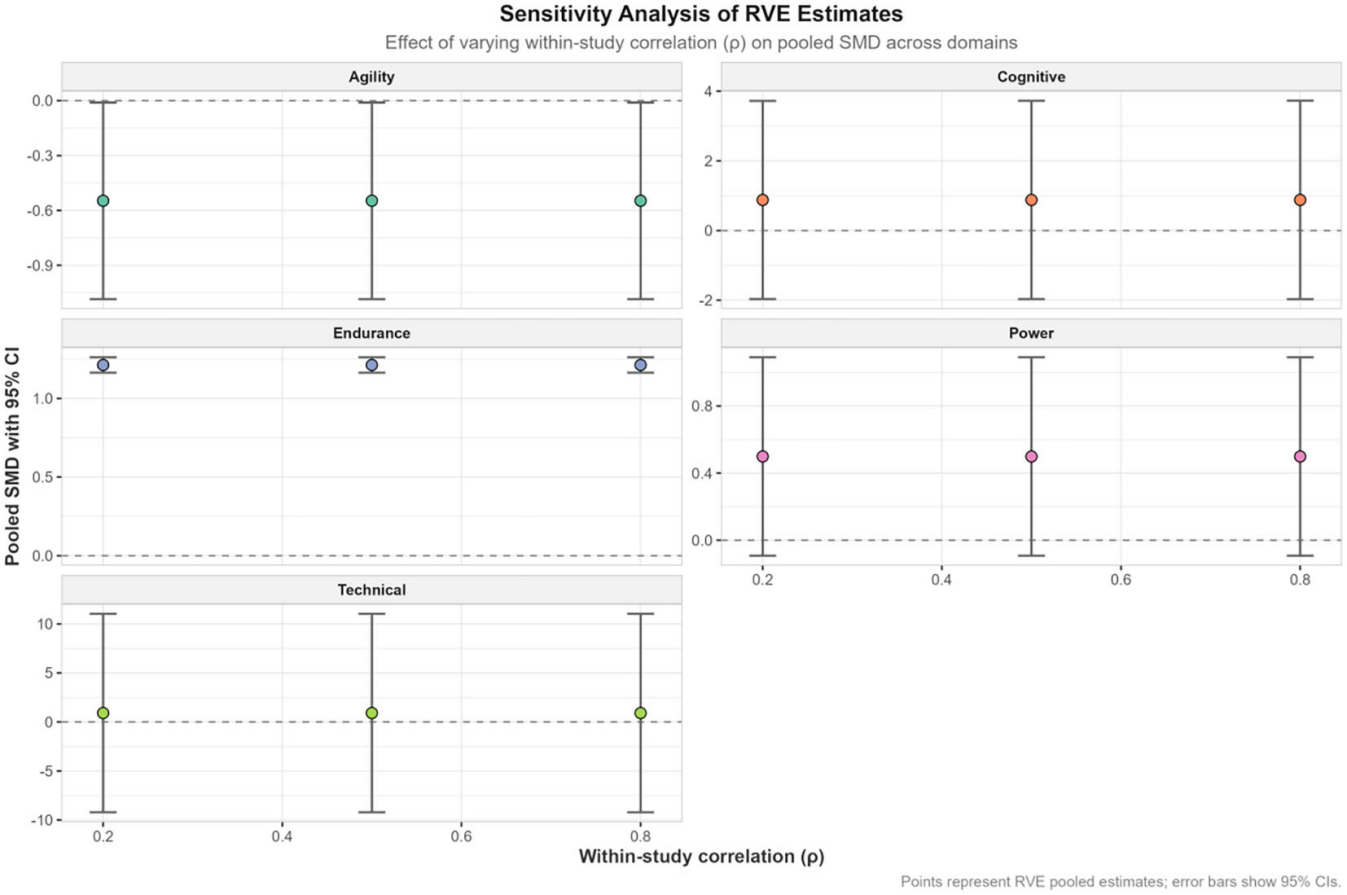
Sensitivity analysis of pooled effect estimates across five performance domains under varying within-study correlation assumptions.

Finally, sensitivity analyses utilizing alternative within-study correlation assumptions (ρ = 0.2, 0.5, 0.8) demonstrated that the pooled SMDs were consistently robust across the entire range of ρ = 0.7 (all domain differences < 0.01; see **Figure 4**). This confirms the robustness of the main findings to the chosen ρ value.

### 3.5 Risk of bias

The overall risk of bias assessment showed that 3 studies (25%) presented some concerns, and 1 study (8.3%) was scored as high risk. The complete report of the risk of bias assessment is available in Supplementary Material **Supplementary Figure 1**.

## 4 Discussion

### 4.1 Main findings

This systematic review and meta-analysis provides the first quantitative synthesis of the effects of daytime napping on athletic performance, specifically in team ball sports athletes. The main findings can be summarized as follows. First, daytime napping exerted a large beneficial effect on endurance performance (*SMD* = 0.96) and a moderate improvement in agility (*SMD* = 0.55). Second, and arguably one of the most compelling findings of this review, the effects of napping on cognitive and technical performance exhibited profound variability and wide confidence intervals. This heterogeneity highlights that cognitive and complex motor tasks respond to napping in a highly individualized manner. Third, nap duration modulated the magnitude of physical effects, with longer naps (>60 min) producing the largest effects on endurance, while brief and moderate naps (≤60 min) consistently conferred benefits for agility.

### 4.2 Physical performance domains and the role of nap duration

Our results suggest a potential duration-dependent trend for the ergogenic benefits of napping on physical performance, with longer naps generally associated with larger point estimates across some physical domains. However, it is imperative to acknowledge the statistical limitations within these subgroup analyses. Specifically, the restricted number of contributing studies in certain subgroups yields wide confidence intervals and low effective degrees of freedom. Therefore, while a duration-dependent pattern is plausible, the current evidence is insufficient to establish a definitive causal dose-response relationship. The most robust finding of this meta-analysis was the large, consistent effect of napping on endurance performance. This domain, comprising measures such as the Yo-Yo intermittent recovery test, showed the lowest heterogeneity *(τ² = 0)*. The observed *SMD* of 0.96 indicates an improvement approaching one standard deviation, which is highly meaningful in competitive settings such as soccer (Altmann et al., 2020). Notably, long naps (>60 min) produced the largest effect on endurance (*SMD =* 1.38), while moderate naps (31–60 min) also yielded substantial improvements (*SMD =* 0.85).

For power and strength performance, the magnitude of the point estimate also increased with nap duration: short naps (≤30 min) produced a small effect (*SMD =* 0.58), moderate naps a small-to-medium effect (*SMD =* 0.29), and long naps a large effect (*SMD =* 0.84). However, the wide confidence intervals crossing zero reflect the limited number of contributing studies (e.g., k = 2 for long naps), precluding definitive conclusions. One possible explanation for the inconclusive findings in the power domain is that explosive power tasks may be more sensitive to sleep inertia, and the included studies varied in the interval between nap awakening and testing. In terms of agility, a consistent moderate improvement was observed. In team ball sports, agility is defined as a rapid whole-body movement with change of velocity or direction in response to a stimulus, representing a critical perceptual-motor coupling (Ardern et al., 2022). This reinforces our decision to categorize agility separately, as its unique requirement for rapid information processing and physical response may be specifically enhanced by napping-induced cognitive alertness. The observed ergogenic effect suggests that daytime napping not only facilitates physical recovery but also enhances the central nervous system’s capacity for rapid information processing and neuromuscular coordination. This finding aligns with the notion that tasks requiring high levels of cognitive engagement and cognitive-motor integration are particularly sensitive to the restorative properties of sleep (Walsh et al., 2021; Büchel et al., 2022).

The physiological mechanisms underlying this duration-dependent trend are intrinsically linked to sleep architecture. Naps of shorter duration (<30 min) typically comprise only light (N1 and N2) non-rapid eye movement (NREM) sleep (Alger et al., 2012; Auger et al., 2015). Moderate naps allow entry into slow-wave sleep (SWS), which is critical for endocrine restitution, such as the pulsatile release of growth hormone that facilitates myofibrillar repair and muscle glycogen resynthesis (Walsh et al., 2021; Dattilo et al., 2011; Charest and Grandner, 2020), while long naps (>60 min) permit a full sleep cycle, including REM sleep. This stage is particularly implicated in the attenuation of central nervous system (CNS) fatigue, restoration of optimal neuromuscular drive, and consolidation of complex motor skills essential for team sports (Stickgold, 2005). Despite these acute performance benefits, it is imperative to interpret the findings of prolonged naps (>60 min) in light of their potential impact on overall circadian organization. While the current meta-analysis highlights the acute ergogenic benefits of napping, habitual or prolonged daytime napping could, under certain circumstances, contribute to rest-activity rhythm fragmentation and weaken the amplitude of the circadian pacemaker. Consequently, while a prolonged nap may be a potent acute countermeasure on match day, practitioners should be cautious about prescribing long naps on a daily, habitual basis, as maintaining a consolidated nocturnal sleep-wake rhythm remains the cornerstone of circadian health and athletic recovery.

### 4.3 The high variability of cognitive and technical performance

In stark contrast to the consistent enhancements observed in physical domains, cognitive and technical performance showed highly variable effects. The substantial statistical uncertainty in these domains represents a critical neurophysiological and methodological finding rather than a mere statistical limitation. This extreme variability can be mechanistically and methodologically explained by the complex interplay of sleep inertia, waking protocols, individual sleep architecture, and diverse assessment instruments.

First, cognitive function and the high-order perceptual-motor coupling required for technical skills are exceptionally vulnerable to sleep inertia—the transient state of impaired performance and reduced vigilance immediately following awakening (Lim and Dinges, 2010). Because cognitive domains and sport-specific tasks vary inherently in their sensitivity to sleep dynamics (Lastella et al., 2021), the lack of standardized waking protocols meant athletes likely performed tasks under varying degrees of incomplete central nervous system arousal. Furthermore, the substantial unexplained heterogeneity in the technical domain (τ² = 0.93) directly stems from fundamental differences in measurement instruments. For instance, simple metrics focusing on a single outcome (e.g., Nishida et al., 2021) may be highly susceptible to ceiling effects (Wang et al., 2009), whereas composite technical indices (e.g., Souabni et al., 2023b) might be significantly more sensitive to subtle cognitive-motor enhancements (Mann et al., 2022).

Second, inter-individual differences in sleep architecture significantly skew outcomes. Brief naps primarily consist of light NREM sleep, which may be insufficient for cognitive-motor restoration, whereas long naps (>60 min) allow entry into REM sleep. REM sleep is essential for the consolidation of complex motor skills and the attenuation of central nervous system fatigue; however, awakening directly from deeper sleep stages exacerbates sleep inertia. Finally, while afternoon napping aligns with the natural post-prandial circadian dip, individual chronotypes dictate the actual restorative efficiency of this window, leading to the highly divergent outcomes observed among team sport athletes.

### 4.4 Comparison with previous literature and methodological considerations

The present meta-analysis confirms and extends previous qualitative reviews. Recent reviews (Mesas et al., 2023; Boukhris et al., 2024; Souabni et al., 2021) and narrative syntheses (Botonis et al., 2021) reported positive effects of napping on performance, with Lastella et al. (2021) recommending nap durations of 20−90 min. Our data refine this guidance by providing domain-specific quantitative effect sizes and demonstrating the superior efficacy of longer naps for endurance.

A major methodological strength of the present meta-analysis is the rigorous, domain-specific taxonomy of athletic performance. Previous systematic reviews and meta-analyses (Mesas et al., 2023) often aggregated divergent tasks—such as maximal vertical jumps and prolonged repeated sprints—into a broad “physical performance” category. Given the high-intensity, intermittent, and open-skill nature of team ball sports, such oversimplification likely masks the true ergogenic effects of napping due to significant statistical heterogeneity. By deconstructing performance into five distinct domains based on specific bioenergetic and neuromuscular demands (Sarmento et al., 2022), our analysis successfully isolated the profound benefits of napping on endurance while uncovering the nuanced, potentially sleep-inertia-confounded effects on explosive power. Furthermore, by providing the first quantitative dose-response synthesis of various nap durations across these isolated domains, this review fills a critical gap in the literature, moving beyond general qualitative advice to offer precise, actionable protocols for athletic recovery. Finally, a broader perspective must be adopted when interpreting these findings within sports science. The ergogenic effects of daytime napping should not be considered in isolation, but rather integrated within the highly interconnected framework of chronotype, circadian rhythms, nocturnal sleep quality and duration, global rest-activity rhythms, and the phenomenon of social jetlag. For athletes, training schedules and societal demands frequently conflict with their endogenous biological timing, leading to social jetlag and consequent sleep disruption. In this paradigm, athletic performance outcomes are fundamentally influenced by multiple dimensions of circadian biology, with napping representing only one functional component of a much more complex biological system (Ciorciari and Lamia, 2025). Future research and practical applications must therefore holistically assess the athlete’s 24-hour sleep-wake ecology rather than treating the daytime nap as a standalone intervention.

### 4.5 Limitations and future directions

Several limitations should be considered when interpreting these findings, which directly inform future research priorities. Foremost is the relatively limited number of studies available for each performance domain; this restriction not only reduced statistical power (notably within the power and technical domains) but also precluded the formal assessment of publication bias (Sterne et al., 2011). Critically, the current literature is predominantly composed of trials with relatively small sample sizes (typically 10 to 25 athletes). In sports science, while small samples are common due to the logistical challenges of recruiting highly trained cohorts and executing rigorous crossover sleep interventions, they inherently increase susceptibility to small-study effects. Consequently, there is a methodological risk that the pooled effect sizes—particularly the large magnitude observed in the endurance domain—may be somewhat inflated, constraining the overall precision of our estimates. Therefore, these quantitative findings should be interpreted as promising initial estimates rather than definitive parameters. Larger-scale randomized controlled trials are urgently needed. Crucially, future designs must integrate physiological and neurobiological biomarkers—such as heart rate variability (HRV) for autonomic regulation, salivary cortisol/testosterone for endocrine status, or electroencephalography (EEG) for precise sleep architecture—alongside standardized objective outcome measures (e.g., countermovement jump, psychomotor vigilance task). Another significant limitation is the lack of detailed reporting regarding the chronotype characteristics of the athletes in the majority of the analyzed studies. Consequently, we were unable to extract sufficient data to perform a subgroup analysis based on chronotype. This must be explicitly acknowledged as a limitation, as the role of chronotype deserves careful consideration. The established relationship between chronotype, napping behavior, and physical performance suggests that an athlete’s chronotype intrinsically influences their natural sleep-wake timing, daily alertness patterns, and the phase of their physical performance fluctuations across the day. Therefore, individual chronotype likely represents an important moderator of the observed ergogenic effects of napping and should be a primary variable controlled for in future randomized trials.

Another significant limitation is the reliance on self-reported sleep duration in several studies. Because self-reported data and standard actigraphy possess limited specificity in distinguishing quiet wakefulness from true neurophysiological sleep, future research should ideally employ full polysomnography (PSG) or validated portable electroencephalography (EEG) to not only verify sleep occurrence but also precisely characterize sleep architecture (e.g., quantification of SWS and REM stages). Furthermore, the included studies predominantly comprised young male athletes. Given that the effects of napping may differ due to physiological and hormonal variations, future research must extend to female athletes across different phases of the menstrual cycle, as well as adolescent and masters athletes, to enhance the generalizability of the evidence base. Moreover, most included studies did not strictly control for or stratify participants by their habitual napping status. It remains unclear whether the observed ergogenic effects represent a true performance enhancement or merely the avoidance of performance decrements associated with nap deprivation in habitual nappers. Finally, ecologically valid studies conducted during tournament play or after cross-timezone travel would clarify whether laboratory-derived effects translate effectively to real-world competitive settings.

### 4.6 Practical implications

It is widely recognized that team-sport athletes frequently accumulate sleep debt due to travel, late-night matches, and anxiety. Notably, the benefits of napping were observed predominantly in athletes with presumed normal nocturnal sleep, indicating that strategic daytime naps confer supplemental advantages even in the absence of prior sleep debt (Mesas et al., 2023). However, for those experiencing partial sleep deprivation, practitioners must acknowledge that while daytime napping is a highly effective acute countermeasure to mitigate alertness and performance decrements, it cannot fully replicate the comprehensive physiological restitution provided by adequate nocturnal sleep.

To translate our findings into actionable protocols, we propose a tailored “Decision Matrix” for team sports based on our newly integrated conceptual framework (see **Supplementary Material** **Supplementary Figure 2**). The optimal nap window lies between 13:00 and 15:30, with 14:00 being the most recommended start time to align with natural circadian rhythms. Crucially, nap duration can be optimized based on the sport’s primary bioenergetic demands, though practitioners must tailor these frameworks to the high contextual fluidity of team sports schedules:

Endurance-Dominant Sports (e.g., Soccer): The current meta-analytic point estimates suggest that longer nap opportunities (>60 min) may offer a distinct recovery advantage, potentially maximizing physiological benefits when the microcycle allows.

Agility and Power-Dominant Sports (e.g., Basketball, Volleyball, Rugby): When scheduling is constrained, brief naps (≤30 min) may serve as a pragmatic alternative, offering initial, time-efficient enhancements, whereas moderate naps (31–60 min) appear to support more consistent improvements in agility.

Cognitive and Technical Demands: Due to the substantial variability in cognitive and technical outcomes, napping protocols targeting skill execution necessitate an individualized approach. Athletes are advised to experiment with individualized nap protocols during training to explore potential personal strategies before considering their implementation in competitive fixtures.

Finally, to combat the disruptive effects of sleep inertia, our framework proposes a structured “Transition Zone.” Practitioners are encouraged to carefully monitor the interval between nap awakening and sport activity; specifically, the pooled physiological evidence suggests that a buffer of at least 60 min is highly advisable to allow core body temperature and sympathetic nervous system activity to elevate, thereby facilitating the dissipation of sleep inertia before competition (Brotherton et al., 2019).

## 5 Conclusions

In summary, this study quantifies the ergogenic potential of daytime napping on athletic performance, specifically in team ball sports athletes, extending previous qualitative reviews by estimating the magnitude of benefits across distinct performance domains. We concluded that daytime napping exerts a large beneficial effect on endurance performance and a consistent improvement in agility performance. For power and strength performance, the point estimate suggested a moderate positive trend, but the wide confidence intervals crossing zero precluded definitive conclusions. Notably, nap duration modulated these effects, with long naps (>60 min) producing the largest improvements for endurance and a positive trend for power, while moderate naps (31–60 min) also conferred substantial benefits for endurance. In contrast, evidence for cognitive performance was limited by substantial heterogeneity and small study numbers, preventing firm inferences from being drawn. For technical performance, the available evidence *(k = 2 studies, df = 1.0)* is insufficient to permit any definitive conclusion. These findings primarily apply to young male team ball sports athletes under conditions of presumed normal nocturnal sleep, indicating that napping confers supplemental performance benefits even when presumed normal nocturnal sleep duration is achieved, though occult sleep micro-debts cannot be entirely ruled out. The results suggest that strategic daytime napping holds promise as a supplemental recovery tool within training and competition routines for team ball sports athletes, provided that protocols remain adaptive to specific performance domains. However, given the limited number of studies, wide confidence intervals, and the clear demographic skew toward young male participants, these recommendations should be viewed as evidence-guided heuristics rather than universal mandates. Consequently, further empirical validation with larger samples, standardized outcome measures, and more diverse populations—including female athletes and other age groups—is warranted to precisely define the boundaries of these practical recovery paradigms.

## Data Availability

The original contributions presented in the study are included in the article/**Supplementary Material**. Further inquiries can be directed to the corresponding author.
