# Supplementary material for: The effect of daytime napping on the athletic performance of team ball sports athletes: systematic review and meta-analysis
Source: Front Physiol. 2026 Jul 22;17:1878224. doi: 10.3389/fphys.2026.1878224 (PMC13439405; doi:10.3389/fphys.2026.1878224)
Supplement: Supplementary file 1 [file SupplementaryFile1.docx]

**Table S1. Search strategy (September 18, 2025) detailed for each database**

**PubMed:**

(nap OR "daytime nap" OR "afternoon nap" OR "power nap" OR "brief sleep" OR siesta) AND (soccer OR football OR basketball OR volleyball OR handball OR rugby OR hockey OR "ice hockey" OR "water polo" OR "team sport*" OR "collective sport*" OR athlete* OR player*) AND (performance OR "athletic performance" OR endurance OR sprint* OR speed OR strength OR power OR "jump height" OR "yo-yo" OR "reaction time" OR accuracy OR fatigue OR "cognitive performance" OR attention OR vigilance OR alertness OR "decision making")

**Scopus:**

TITLE-ABS-KEY TITLE-ABS-KEY (nap OR "daytime nap" OR "afternoon nap" OR "power nap" OR "brief sleep" OR siesta) AND TITLE-ABS-KEY (soccer OR football OR basketball OR volleyball OR handball OR rugby OR hockey OR "ice hockey" OR "water polo" OR "team sport*" OR "collective sport*" OR athlete* OR player*) AND TITLE-ABS-KEY (performance OR "athletic performance" OR endurance OR sprint* OR speed OR strength OR power OR "jump height" OR "yo-yo" OR "reaction time" OR accuracy OR fatigue OR "cognitive performance" OR attention OR vigilance OR alertness OR "decision making")

**Web of Science:**

TS=(nap OR "daytime nap" OR "afternoon nap" OR "power nap" OR "brief sleep" OR siesta) AND TS=(soccer OR football OR basketball OR volleyball OR handball OR rugby OR hockey OR "ice hockey" OR "water polo" OR "team sport*" OR "collective sport*" OR athlete* OR player*) AND TS=(performance OR "athletic performance" OR endurance OR sprint* OR speed OR strength OR power OR "jump height" OR "yo-yo" OR "reaction time" OR accuracy OR fatigue OR "cognitive performance" OR attention OR vigilance OR alertness OR "decision making")

**Cochrane Library:**

(nap OR "daytime nap" OR "afternoon nap" OR "power nap" OR "brief sleep" OR siesta) AND (soccer OR football OR basketball OR volleyball OR handball OR rugby OR hockey OR "ice hockey" OR "water polo" OR "team sport*" OR "collective sport*" OR athlete* OR player*) AND (performance OR "athletic performance" OR endurance OR sprint* OR speed OR strength OR power OR "jump height" OR "yo-yo" OR "reaction time" OR accuracy OR fatigue OR "cognitive performance" OR attention OR vigilance OR alertness OR "decision making") in Title Abstract Keyword - (Word variations have been searched)

**SportDiscus:**

AB (nap OR "daytime nap" OR "afternoon nap" OR "power nap" OR "brief sleep" OR siesta) AND AB (soccer OR football OR basketball OR volleyball OR handball OR rugby OR hockey OR "ice hockey" OR "water polo" OR "team sport*" OR "collective sport*" OR athlete* OR player*) AND AB (performance OR "athletic performance" OR endurance OR sprint* OR speed OR strength OR power OR "jump height" OR "yo-yo" OR "reaction time" OR accuracy OR fatigue OR "cognitive performance" OR attention OR vigilance OR alertness OR "decision making")

**Table S2. Excluded studies by reason for exclusion (n = 13)**

**Inappropriate study design** (n=8)

1. Ajjimaporn, A., Ramyarangsi, P., and Siripornpanich, V. (2020). Effects of a 20-min Nap after Sleep Deprivation on Brain Activity and Soccer Performance. Int J Sports Med 41, 1009–1016.

2. Ammar, A., Boukhris, O., Hsouna, H., Ben Dhia, I., Trabelsi, K., Ali Gujar, T., et al. (2021). The effect of a daytime 60-min nap opportunity on postural control in highly active individuals. bs 38, 683–691.

3. Boukhris, O., Suppiah, H., and Driller, M. (2025). The Influence of 25‐ and 90‐Min Afternoon Nap Opportunities on Subsequent Nighttime Sleep in Student Athletes. Journal of Sleep Research, e70120

4. Pérez, C. A. (2017). Iron Deficiency And Physical Performance In Elite Female Soccer Players: 2074 Board #87 June 1 3 30 PM - 5 00 PM. Medicine & Science in Sports & Exercise 49, 570.

5. Bolin, D. J. (2019). Sleep Deprivation and Its Contribution to Mood and Performance Deterioration in College Athletes. Curr Sports Med Rep 18, 305–310.

6. Waterhouse, J., Atkinson, G., Edwards, B., and Reilly, T. (2007). The role of a short post-lunch nap in improving cognitive, motor, and sprint performance in participants with partial sleep deprivation. Journal of Sports Sciences 25, 1557–1566.

7. O’Donnell, S., Beaven, C., and Driller, M. (2018). The Influence of Match-Day Napping in Elite Female Netball Athletes. INTERNATIONAL JOURNAL OF SPORTS PHYSIOLOGY AND PERFORMANCE 13, 1143–1148.

8. Mah, C. D., Mah, K. E., Kezirian, E. J., and Dement, W. C. (2011). The Effects of Sleep Extension on the Athletic Performance of Collegiate  Basketball Players. Sleep 34, 943–950.

**Subjects not meeting criteria** (n=2)

1. Ammar, A., Boukhris, O., Hsouna, H., Ben Dhia, I., Trabelsi, K., Gujar, T., et al. (2021). The effect of a daytime 60-min nap opportunity on postural control in highly active individuals. BIOLOGY OF SPORT 38, 683–691.

2. Kurtoğlu, A., Eken, Ö., Aydın, E., Çar, B., and Nobari, H. (2024). The impact of strategic napping on peak expiratory flow and respiratory function in young elite athletes. BMC Sports Sci Med Rehabil 16, 41.

**Insufficient data provided** (n=2)

1. Teece, A. R., Beaven, M., Huynh, M., Argus, C. K., Gill, N., and Driller, M. W. (2023). Nap to perform? Match-day napping on perceived match performance in professional rugby union athletes. International Journal of Sports Science & Coaching 18, 462–469.

2. Souabni, M., Souabni, M., Hidouri, S., Ammar, A., Younes, M., Hammouda, O., et al. (2024). Napping and heart rate variability in elite athletes. BIOLOGY OF SPORT 41, 213–221

**Special physiological state** (n=1)

1. Hsouna, H., Boukhris, O., Ammar, A., Trabelsi, K., Clark, C. C. T., Abdessalem, R., et al. (2025). Effects of a 40-min nap on performance following evening exercise before, during, and after Ramadan fasting. Research in Sports Medicine 33, 224–240.

**Figure S1:** The overall risk of bias for each

**Figure S2:** Preliminary conceptual framework for strategic daytime napping
